# Prevalence of depression in Parkinson’s disease patients in Ethiopia

**DOI:** 10.1186/s40734-014-0010-3

**Published:** 2014-12-12

**Authors:** Dawit Kibru Worku, Yared Mamushet Yifru, Douglas G Postels, Fikre Enquselassie Gashe

**Affiliations:** Addis Ababa, Ethiopia; Department of Neurology, Addis Ababa University, Addis Ababa, Ethiopia; International Neurologic and Psychiatric Epidemiology Program, Michigan State University, Michigan, USA; Department of Community Health, Addis Ababa University, Addis Ababa, Ethiopia

**Keywords:** Parkinson’s disease, Depression, Non-Motor symptoms

## Abstract

**Background:**

Parkinson’s disease (PD) is associated with cognitive and psychiatric disturbances including depression, anxiety, psychotic symptoms and sleep disturbances. These psychiatric manifestations have a negative impact on disease course and the medical management of PD patients. Major depression has a greater negative impact on patients’ quality of life than abnormal motor function, and is associated with faster cognitive decline and progression of motor deficits. Thus, the objective of this study was to determine the prevalence and pattern of depression in PD outpatients in Ethiopia. We determined the age range in which depression in PD patients is most common, the most common symptoms of depression, and the epidemiologic confounders associated with depression in PD patients.

**Methods:**

We conducted a cross-sectional point prevalence study of all PD patients attending the follow-up clinics of the departments of neurology at Black Lion Teaching and Zewuditu Memorial Hospitals in Addis Ababa, Ethiopia, from May 2013 to August 2013. We collected information using a structured questionnaire which assessed demographic information, clinical history, and neurologic function.

**Result:**

Of the 101 patients surveyed, the prevalence of depression was 58/101(57.4%). Of these patients, 1 of 58(1.7%) was on antidepressant medications. These low proportions likely indicate a low index of suspicion and under treatment of depression in PD outpatients.

**Conclusion:**

In Ethiopian PD outpatients, depression is under recognized and undertreated. We recommend routine use of screening tools. In those who screen positive for depression, treatment is warranted. Further studies are needed to confirm these findings, and to increase our understanding of specific signs and symptoms of depression in the context of PD.

**Electronic supplementary material:**

The online version of this article (doi:10.1186/s40734-014-0010-3) contains supplementary material, which is available to authorized users.

## Background

Worldwide, Parkinson’s disease (PD) is the most common progressive neurodegenerative disorder of adulthood and is characterized by bradykinesia, resting tremor, muscular rigidity, shuffling gait, and flexed posture. PD may be accompanied by a variety of non-motor symptoms, including autonomic, sensory, sleep, cognitive, and psychiatric disturbances [1]-[5].

### Psycihiatric Co-morbidity in Parkinson’s disease

In addition to motor abnormalities, PD is associated with cognitive and psychiatric disturbances including depression, anxiety, psychotic symptoms and sleep disturbances [6],[7]. Psychiatric co-morbidities have a negative impact on the course and management of PD patients. Major depression has a greater negative impact on patients’ quality of life than impaired motor function [8]-[10] and accounts for more rapid cognitive decline and progression of motor deficits [11]-[14].

Early recognition and treatment of co-morbid depressive disorders is critical for comprehensive patient management. Failure to diagnose psychiatric co-morbidities in PD patients is common. In a study of 101 PD patients done in Baltimore, Maryland, USA, the treating neurologists failed to recognize depression, anxiety and fatigue in more than half of patients [15]. Standardized testing showed depression in 44% of patients, anxiety in 39%, fatigue in 42%, and sleep disturbance in 43%. The prevalence of these conditions as identified by the treating neurologist was lower: 21% with depression, 19% with anxiety, 14% with fatigue and 39% with sleep disturbance. The diagnostic sensitivity for the treating neurologists was 35% for depression, 42% for anxiety, 25% for fatigue, and 60% for sleep disturbance [15].

### Depression screening tools

Depression screening tools should be both sensitive but also specific to the broad differential diagnosis of depressed mood in PD. They should differentiate between a major depressive episode, adjustment disorder, mood disorder, dementia, “non-motor fluctuation”, and transient mood changes in relation to DBS. Although not a substitute for a diagnostic evaluation, scales should distinguish normal emotional variability from symptoms that reflect major depression or a disabling non-major depressive syndrome. Clinician-rated depression scales include the 24-item and 17-item Hamilton Depression Rating Scale, the Montgomery-Åsberg Depression Rating Scale, and the Unified Parkinson’s Disease Rating Scale Depression item. Self-reported scales include the Beck Depression Inventory–Version I, the 30-item and 15-item Geriatric Depression Scale, and Quick Inventory of Depressive Symptomatology. Clinician-Rated scales have been shown to be valid in PD [16]. The GDS-15 and Patient Health Questionnaire-9 have also been investigated as diagnostic instruments in PD [17].

A comparison study of nine scales to detect depression in PD showed the QIDS-C16 scale were among the valid screening when PD-specific cutoff scores are used [18]. To determine if the QIDS-C16 measure depression in a manner consistent with the most widely used assessments, Rush et al. [19] examined the relationship between QIDS scores, and the Hamilton Rating Scale for Depression 17 item version (HRSD17) and Beck Depression Inventory (BDI) scores in a sample of 434 outpatients with major depressive disorder and 103 normal controls. QIDS total score was comparable to those obtained by the HRSD17 and BDI, with Pearson product moment correlations of 0.95 between the IDS-C30 and HRSD17. The correlation between the BDI21 and the IDS-C30 was 0.86. The correlation between the BDI21 and the HRSD17 was 0.85.

Providing evidence that the IDS and QIDS are measuring depressive symptoms in the same manner, Trivedi et al. [20] found robust correlations between the QIDS-C16 and IDS-C30 total scores for out-patients with MDD (c = 0.82, n = 544) and Bipolar Disorder (BD) (c = 0.81, n = 402). As the Quick Inventory of Depressive Symptomatology Clinician-Rated – 16 scale includes the core components of the DSM-IV TR criteria for depression, we have chosen it for use in this research.

**Figure 1:**
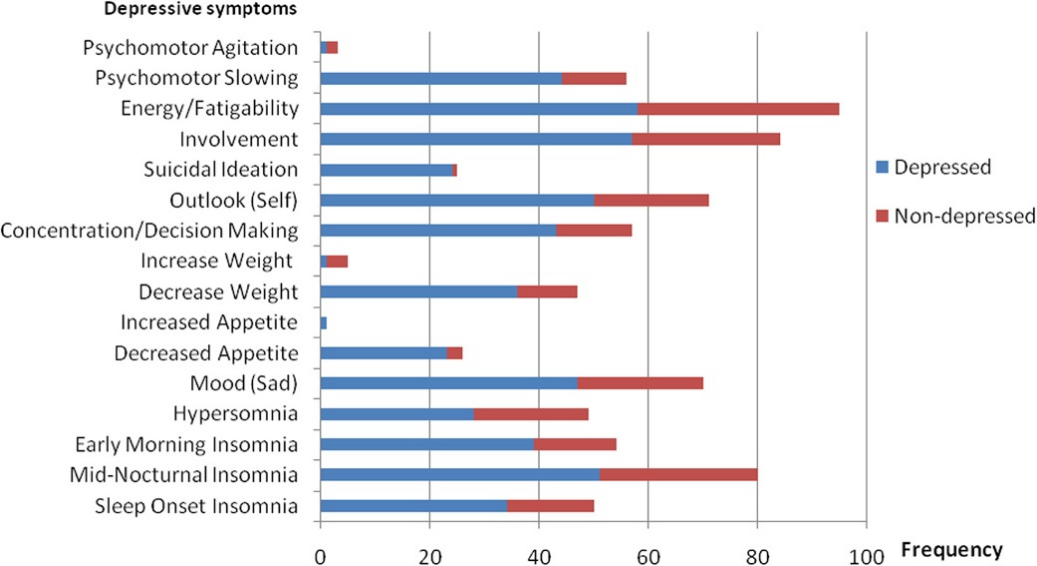
**Frequency distributions of depressive symptoms of study subjects, Black Lion Hospital and Zewuditu Memorial Hospital.**

Trivedi et. al found that by using the QIDS-C16 and IDS-C30, that within the patient population studied (drawn from 19 regionally and ethnically diverse clinics as part of the Texas Medication Algorithm Project, Texas, USA) the severity of depression varied greatly: 43(42.6%) had mild, 14(13.9%) had moderate, 1(0.99%) had severe depression, and 43(42.6%) had no depression. There was no patient who was very severely depressed (Figure 1). In other studies co-morbid depression prevalence rates vary, ranging from 7% to 90%, with a general consensus that depression in some form (i.e., either major or non-major depression) appears to occur in approximately 40% of PD patients [14],[21].

PD-specific cutoff scores (i.e., >12) increase the ability of QIDS to detect depression in PD to: sensitivity of 0.81, specificity of 0.79, positive predictive value of 0.73, and negative predictive value of 0.86. But it’s not a recommendation for this cutoff score to be used in clinical practice [18].

Depression and PD are associated. Patients with PD are at higher risk of developing depression than the general population and patients with depressive disorder are at greater risk of developing PD. Indeed, two population-based retrospective cohort studies have shown that, compared with controls of healthy subjects [22] or medical patients [23], patients with PD were more likely to have experienced a history of depression before their neurological diagnosis was made (odds ratios 2.2 and 2.4, respectively (95% CI = 2.1–2.7)).

There is little information available on the prevalence of depression in PD patients in Ethiopia. A study of existing indexed literature indicates that comparatively little PD-related research has been published from Africa [24]. However, because Africa is experiencing a demographic transition, the population will become much older by 2015 (increase in the percentage of persons aged 65 and over) [25], and diseases predominantly affecting older persons, such as PD are expected to become more common. This is also true for Ethiopia. Therefore, epidemiologic data are needed for effective planning of medical services in African countries including Ethiopia, and to compare genetic, environmental, and clinical aspects of PD in Africa with those of other continents.

Depression in PD often begins late in life, in contrast to primary major depression, which is more likely to appear before the age of 40. Analogous to other neurologic disorders where depression is co-morbid (epilepsy, multiple sclerosis and dementia) mood disturbances can antedate the motor manifestations of disease. This occurs in 12-37% of PD patients [21]. Dysphoric symptoms, including irritability, sadness, and pessimism, are more frequent presenting symptoms in PD patients compared to patients with primary mood disorders. Feelings of failure, guilt, and self-blame are less frequent in those with depression and co-morbid PD. Though suicidal ideation is more frequent in PD patients with co-morbid mood disorders compared to patients with primary depression, PD patients are less likely to commit suicide [21].

Depression is also a key determinant of poor health-related quality of life in PD patients and is associated with reduced function, cognitive impairment and increased stress for individuals with PD.

PD patients were significantly more depressed than other disabled patients matched for extent of functional impairment [26]. In studies of the relationship between depression and severity of functional impairment most have identified a modest correlation between mood abnormalities and functional deterioration [21]. Huber et al. reported that depressed patients with PD had a significantly more advanced stage of disability than nondepressed patients with Parkinson’s disease [27]. Understanding depression in patients with PD is, therefore, critical to achieve comprehensive care for patients with this disease. Screening and the early identification of depression offer a crucial opportunity to alter the course of the disease and improve quality of life.

The main objective of the study is to determine the prevalence and pattern of depression in PD patients seen in the outpatient clinics of Black Lion and Zewuditu Memorial Hospitals, Addis Ababa, Ethiopia.

## Methods

A Cross-sectional point prevalence survey was conducted between June to November 2013 in two governmental referral hospitals; Black Lion Teaching Hospital and Zewuditu Memorial Hospital, Addis Ababa, Ethiopia. All patients diagnosed with PD and attending the Neurology follow-up clinics of Black Lion Hospital or Zewuditu Memorial Hospital were the source population.

Patients were included if they had previous diagnosis of PD, were age ≥18 years old, had clinical symptomatology in agreement with the UK Parkinson’s Disease Society Brain Bank Clinical Diagnostic Criteria for the diagnosis of PD, and informed consent granted for study participation. Patients were excluded if they had Parkinsonism due to other causes or if they were unable to give consent. All patients fulfilling the inclusion criteria from June 2013 to November 2013 in the study area was included.

Clinical data was collected using a structured questionnaire in English. This questionnaire assessed: demographic data; a detailed clinical history including symptoms of PD and their duration; medication history.

A complete clinical and neurologic examination was performed. Nervous system examinations include full assessment of cranial nerves, deep tendon reflexes, Babinski sign, tone, motor and sensory functions, cerebellar signs, postural instability, gait and stance, and abnormal movements.

The 16 item Quick Inventory of Depressive Symptomatology (QIDS-C16) screen was administered and a score for each patient was calculated. Subjects were interviewed by clinicians to complete the QIDS-C16. The symptoms were familiar to clinicians, as the individual items are defined by the constructs represented in the DSM-IV criteria for MDD. Each item is interval-scaled from 0 to 3; 0 indicates absence of the symptom during the last 7 days. The anchors are intended to help raters represent the frequency and intensity associated with each symptom. Total QIDS-C16 total scores range from 0 to 27. The total score was obtained by adding the scores for each of the nine symptom domains of the DSM-IV MDD criteria: depressed mood, loss of interest or pleasure, concentration/decision making, self-outlook, suicidal ideation, energy/fatigability, sleep, weight/appetite change, and psychomotor changes. Sixteen items were used to rate the nine criterion domains of major depression: four for sleep disturbance (early, middle, and late insomnia plus hypersomnia); two for psychomotor disturbance (agitation and retardation) and four for appetite/weight disturbance. Only one item was used to rate the remaining six domains (depressed mood, decreased interest, decreased energy, worthlessness/guilt, concentration/decision making, and suicidal ideation). Each item was rated 0–3. For symptom domains that require more than one item, the highest score of the item relevant for each domain was taken (e.g. , if early insomnia is 0, middle insomnia is 1, late insomnia is 3, and hypersomnia is 0, the sleep disturbance domain was rated 3). Severity of depression was defined by Total QIDS-C16 score: None for 0 – 5, Mild for 6 – 10, Moderate for 11 – 15, Severe for 16 – 20, and Very Severe for 21 – 27.

Piloting of the questionnaire was done in a sample of ten PD patients attending Black Lion Hospital Neurology Clinic before the starting of data collection for the research. These subjects were not included in the study results. Findings from the pre-test were utilized in modifying questions on the standard questionnaire. Questions that were difficult for subjects to understand were reformulated and repiloted until answers were considered internally valid.

Interviews and data extraction was performed by the principal investigator. Data was checked manually and cleaned. Before processing the data was coded and cross checked for completeness.

Analysis was performed using SPSS/PC version 20.0 software packages for statistical analysis (SPSS, INC, Chicago, IL). Descriptive summaries were employed to describe socio-demographic and clinical characteristics. Appropriate measures of central tendency, frequency distribution and cross tabulation were conducted. Odds ratios and 95% confidence intervals were calculated. A p value less than 0.05 were considered a statistically significant association between assessed variables. Due to the exploratory nature of our study we did not correct for multiple comparisons.

Protocol approvals were obtained from the ethical review Committee of the Department of Neurology and the Institutional Review Board (IRB) and Research and Publication Committee of the College of Health Sciences of Addis Ababa University. Informed patient consent was sought before study enrollment. Patients received standard therapy for PD and co-morbid mood disorders regardless of whether they consented to study enrollment. Patient data was deidentified during subsequent analysis and dissemination.

## Results and discussion

One hundred one subjects with PD consented to study participation. Ninety-nine (98%) were right handed and 70/101 (69.3%) were male (Table 1). Depression was diagnosed in 58/101 (57.4%) patients. The prevalence of depression in women was 58.1% and in men 57.1%. Almost half [42/101(41.6%)] of the patients had no formal education. Twelve (20.7%) of depressed patients were employed while 14 (32.7%) of the non-depressed patients were employed. This was not significantly associated (P-value = 0.18, 95% CI = 0.75-4.55).

**Table 1:** **Frequency distributions of sociodemographic characteristics of study subjects, Black Lion Hospital and Zewuditu Memorial Hospital**

Variables	Depressed		Not depressed		
	Frequency	Proportion	Frequency	Proportion	P-value
**Handedness**					
Right	56	96.6%	43	100	
Left	2	3.4%	-	-	0.5
Total	58		43		
**Age**					
<50	11	19.0%	8	18.6	
50-59	9	15.5%	10	23.3	
60-69	17	29.3%	17	39.5	
70-79	17	29.3%	7	16.3	
80 and above	4	6.9%	1	2.3	
Total	58		43		
Mean ± SD	63.94 ± 12.06		60.13 ± 9.20		0.087
**Gender**					
Female	18	31.0	13	30.2	1.0
Male	40	69.0	30	69.8	
Total	58		43		
**Marital status**					
Married	39	67.2	36	83.7	
Separated/divorced	6	10.3	-	-	0.03
Widowed	13	22.4	7	16.3	0.33
Total	58		43		
**Employment**					
Employed	12	20.7	14	32.6	0.25
Unemployed	46	79.3	29	67.4	
Housewife	10	21.7	7	24.1	1.0
Retired	20	43.5	14	48.3	0.84
Out of job	16	34.8	8	27.6	0.81
Total	58		43		
**Education**					
No formal education	29	50.0	13	30.2	
Primary	18	31.0	10	23.3	0.80
Secondary	6	10.3	12	27.9	0.02
More than secondary	5	8.6	8	18.6	0.06
Total	58		43		
**Monthly income (USD)**					
<300	33	56.9	12	27.9	
300-600	14	24.1	13	30.2	0.08
600-900	5	8.6	7	16.3	0.08
>900	6	10.4	11	25.6	0.01
Total	58		43		
**Recent major life events**					
Yes	4	6.9	1	2.3	0.40
No	54	93.1	42	97.7	
Total	58		43		
**Previous history of depression**					
Yes	2	3.4	-	-	1.0
No	56	96.6	43	100	
Total	58		43		
**First degree family history of depression**					1.0
Yes	3	5.2	2	5.2	
No	55	94.8	41	94.8	
Total	58		43		
**Age at onset**					
<50	15	25.9	15	34.9	0.44
50-59	20	34.5	12	27.9	
60-69	12	20.7	13	30.2	0.30
>70	11	19.0	3	7.0	0.33
Total	58	100	43	100	
**Medication(s) taken/taking**					
Carbidopa-Levodopa	54	40.3	35	49.3	0.12
Trihexyphenidyl	29	21.6	19	26.8	0.69
Antidepressant	1	1.7	2	4.7	0.57

Forty-five (44.55%) had annual income of <300 USD and only 17(16.83%) of the study patients had a monthly income of >900 USD.

Of the total study population 5(4.95%) had a recent major life event, 2(1.98%) had a previous history of depression, 5(4.95%) had a first degree family history of psychiatric disorder, and 6(5.94%) had a family history of PD.

All subjects had bradykinesia, 94(93.1%) of them had muscular rigidity, and 92(91.1%) had resting tremor. Approximately half [50/101(49.5%)] had postural instability. Almost all subjects [100(99%)] had a progressive disorder and very few [7(6.9%)] had severe levodopa-induced chorea. Most of the subjects 89/101 (88.1%) were taking Levodopa-Carbidopa (250 mg-25 mg) combination, 48(47.5%) were on Trihexiphendyl, only 3(2.97%) were on antidepressant (Amitriptyline, Flouxitine, and Serteraline each). Thirteen (29.7%) patients had co-morbid medical condition(s) including hypertension, diabetes mellitus, and bronchial asthma. Only 23(22.8%) patients had previous imaging.

**Figure 2:**
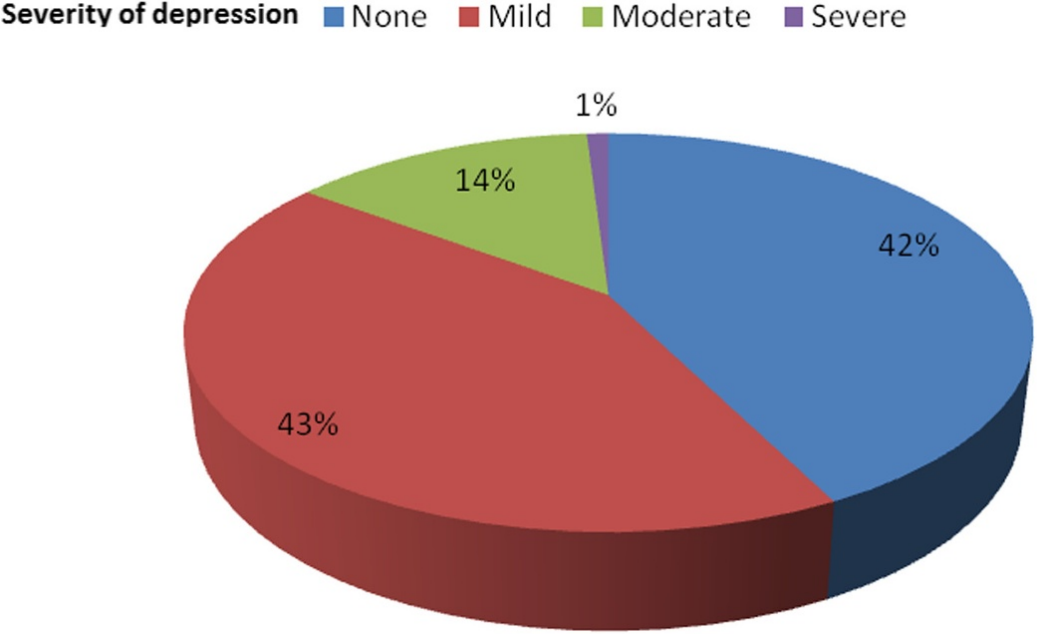
**Frequency distributions of severity of depression of study subjects, Black Lion Hospital and Zewuditu Memorial Hospital.**

Fifteen percent of the study population was classified as having moderate to severe depression (Figure 2). The most common depression symptoms of the screen positive patients were loss of energy, decreased social involvement, and mid-nocturnal insomnia. Increased appetite, increased weight, and psychomotor agitation were among the rare manifestation of depression in the study population.

One third (34.7%) of the patients were diagnosed with PD between the age of 55–64, 27(26.7%) at the age 65 and above, 24(23.8%) at the age 45–54, and only 15(14.9%) at age below 45 years; with mean + SD of 57.10 + 10.84.

Of all the variables evaluated, only annual income was significantly associated (P-Value = 0.01; 95% CI = 0.2(0.06-0.66)) with having depression in the present study (Table 2).

**Table 2:** **Depression Vs Parkinson’s disease characteristics of study subjects, Black Lion Hospital and Zewuditu Memorial Hospital**

Variables	Depressed	Non-depressed	Crude OR	P-value
**Handedness**				
Right	56	43	ref	0.51
Left	2	-	undetermined	
Total	58	43		
**Age**				
<50	11	8	1.38(0.44-4.27)	0.78
50-59	9	10	0.9(0.30-2.77)	1.0
60-69	17	17	ref	0.18
70-79	17	7	2.43(0.80-7.35)	0.35
80 and above	4	1	4.0(0.40-39.58)	
Total	58	43		
**Gender**				
Female	18	13	1.04(0.44-2.45)	1.0
Male	40	30	ref	
Total	58	43		
**Marital status**				
Married	39	36	ref	
Separated/divorced	6	-	undefined	0.03
Widowed	13	7	1.71(0.62-4.78)	0.33
Total	58	43		
**Employment**				
Employed	12	14	0.54 (0.22-1.33)	0.25
Unemployed	46	29	ref	
Total	58	43		
**Education**				
No formal education	29	13	ref	
Primary	18	10	0.80(0.29-2.22)	0.80
Secondary	6	12	0.22(0.07-0.73)	0.02
More than secondary	5	8	0.28(0.08-1.02)	0.06
Total	58	43		
**Annual income(USD)**			ref	
<300	33	12	0.4(0.14-1.07)	0.08
300-600	14	13	0.26(0.07-0.98)	0.08
600-900	5	7		
>900	6	11	0.2(0.06-0.66)	0.01
Total	58	43		
**Recent major life events**				
Yes	4	1	3.11(0.34-28.88)	0.39
No	54	42	ref	
Total	58	43		
**Previous history of depression**				
Yes	2	-	undefined	0.5
No	56	43	ref	
Total	58	43		
**First degree family history of depression**				
Yes	3	2	1.12(0.18-7.00)	1.0
No	55	41	ref	
Total	58	43		
**Age at onset**				
<50	15	15	0.6(0.22-1.65)	0.44
50-59	20	12	ref	
60-69	12	13		
>70	11	3	0.55(0.19-1.60)	0.30
Total	58	43	2.2(0.51-9.51)	0.33
**Medication(s) taken/taking**				
			ref	0.12
Carbidopa-Levodopa			0.32(0.09-1.16)	
Yes	54	35		
No	4	8		
Trihexyphenidyl				
Yes	29	19	1.26(0.57-2.79)	0.69
No	29	24	ref	
Antidepressant				
Yes	1	2	0.36(0.03-4.10)	0.57
No	57	41	ref	
**Co-morbid medical condition(s)**				
Yes	21	9	0.19-1.16	0.12
No	37	34	ref	
Total	58	43		

Depressive symptoms have been recognized to be a major determinant of health-related Quality of life in PD. There is evidence that depression in patients with PD is associated with more severe cognitive and functional impairments, faster progression of illness, worse quality of life and higher burden for caregivers. Several rating scales for screening or assessing the severity of depression are available [28]. A comparison study of nine scales to detect depression in PD showed the QIDS-C16 scale were among the valid screening when PD-specific cutoff scores are used [18].

In Tekle-Haimanot’s study PD was seen more frequently in males (7:3) and the commonest decade was 61–70 (25/70 cases) which are comparable to our finding, male to female ratio of 7:3 and with an age group of 60–70 respectively. The mean age at onset of symptoms was 54.6 years, which was lesser than our finding, 57.1 years [29]. In Zahodne et al’s USA study the male to female ratio was comparable but the age at onset was 59.71 years. These differences may be due to the difference among the life expectancies of the study areas and periods [30].

**Figure 3:**
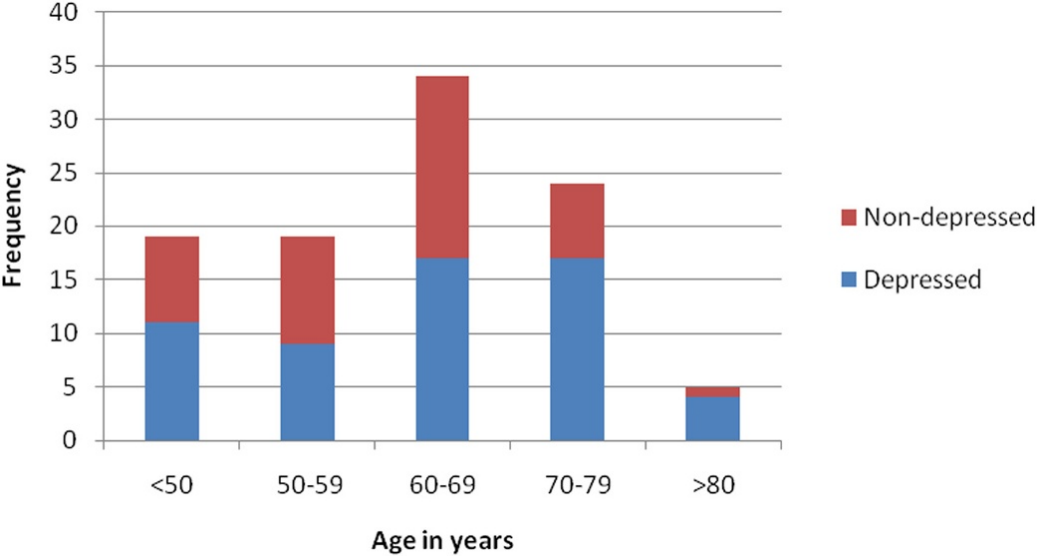
**Frequency distributions of different age groups of study subjects, Black Lion Hospital and Zewuditu Memorial Hospital.**

One third of the patients in the present study were in the age range between 60–69 years. The mean age (years) + SD for depressed patients and non depressed patients were 63.94 + 12.06 and 60.13 + 9.20, respectively (Figure 3). Their mean difference was not significantly associated (t = 1.73, df = 99, P-value = 0.087, 95% CI = −0.56-8.18).

A majority of studies have found no relationship between depression and the patient’s current age or the patient’s age at onset of the PD [21]. This was true in the present study. The boundary age for early-onset versus late-onset Parkinson’s disease has varied among studies, perhaps contributing to the lack of agreement regarding the importance of this variable as a risk factor for depression. Conclusions based on the available data regarding the potential role of age at onset as a contributing factor to the vulnerability to depression would be premature.

The duration of PD might be expected to influence the rate of depression, but most investigators have found no relationship between length of illness and mood changes [21]. It is also in agreement with the present study.

Several studies have found a higher rate of depression among women with PD, but others have failed to identify any relationship between gender and depression [21]. Being female may be a risk factor for depression in PD, but a consensus is lacking and present study also does not show any gender association.

Recent major life events (death of spouse or children, divorce, homelessness, etc), previous history of depression, and family history of depression were among the major risk factor for developing depression [31]. In the present study, 5(4.95%) of them had recent major life event, 2(1.98%) had previous history of depression, and 5(4.95%) had first degree family history of psychiatric disorder. None of these were significantly associated with increased risk of developing depression in the present study. This may be due to relatively small number of PD patients who had these risk factors. In addition, 30(29.7%) study patients had co-morbid medical condition(s). Different studies show having co-morbid medical conditions predispose to depression which was not significantly associated in the present study [32].

Neuroimaging studies such as CT and MRI are also usually unhelpful in making a diagnosis of PD, because they are generally normal or show only incidental abnormalities. On the other hand neuroimaging abnormalities can be useful in suggesting alternative diagnoses such as Progressive Supranuclear Palsy or Multiple System Atrophy [33]. In our study only 23(22.8%) patients had previous imaging. This may affect the definitive diagnosis of PD since having imaging may exclude alternative causes of Parkinsonism.

Our study population differed from those in previous published evaluations of PD-depression associations. In our study only 25.74% of the study patients were employed which was less than from a W.B.P. Matuja’s study. This may be due to the retirement age of the Ethiopian civil servant is currently age >60 which is lesser than our study patients mean(62.33), and also the two studies have different age structure with a mean of 61.50 in W.B.P. Matuja’s study [34].

Almost half of our study patients 43(42.57%) have annual income of <300 USD which is less than the national per capita income (410 USD) [35]. Out of the depressed patients 32(56.1%) have annual income <300 USD and over all high annual income is significantly associated with depression in our study with OR of 0.2(0.06-0.66) (Table 2). The association of psychopathology of depression with poverty has been repeatedly demonstrated in other studies [36].

In contrast to the findings in the present study, depression was conspicuously absent in previously published reports of PD patients in W.B.P. Matuja’s study. These differences in results may be due to variations in depression screening tools and the small sample sizes of all studies to date [34]. In the Zahodne et al. study, of the 95 patients surveyed, 27 (28%) had depressive episode [30].

Several studies have assessed the intensity of depressive symptoms in Parkinson’s disease by using clinical criteria to distinguish between major depressive episodes and dysthymia or by applying rating scales to differentiate mild from moderate-to-severe syndromes. In Cummings review, on average, slightly more than half (54%) of depressed patients with Parkinson’s disease met criteria for a major depressive episode (moderate-to-severe symptoms) and slightly less than half (45%) had dysthymia or minor depressions (mild symptoms). These findings are in agreement with the present study, fifteen percent of the study population was classified as having moderate to severe depression (Figure 2). The proportions varied considerably among studies, again suggesting the influence of considerable selection biases in different study populations. Major and minor syndromes differ in several respects in addition to severity: minor depressions are more likely to remit [37] and are more closely related to disability [38].

If PD-specific cutoff scores (i.e., >12) are used to maximize the sum of sensitivity and specificity, the prevalence of depression is 40.59% (41) in our study. This is comparable to Cummings et al’s review [21].

In PD patients, depressive symptoms affect all domains, including physical, affective, and cognitive. In a previous study examining the symptom profile of depressed PD patients, depressed mood, tension, loss of interest, and loss of concentration were the most common depressive symptoms. Feelings of guilt, self-blame, appetite disturbance, and suicidal ideation were not as common [39]. Symptoms that are common to both depression and idiopathic PD include motor slowing, bradyphrenia, sleep and appetite disturbance, weight loss, loss of interest and concentration, and reduced libido [40]. Another previous study found that low mood, anhedonia, and lack of interest constituted the most prominent symptoms in depressed PD patients, and that reduced appetite and early morning awakening are two somatic items that discriminate between PD patients with and without depression [41].

Sleep disturbances, among other non-motor symptoms, have been reported in 60–90% PD patients and are unrecognized in over 40% of patients with PD, clinicians should actively and routinely inquire about sleep patterns during consultation [42]-[44]. It is same in the present study with all the depressed patients had sleep changes.

There are significant difference in the major depressive symptoms (i.e., mood, sleep changes, self outlook, and suicidal ideation) among the depressed PD patients of the present study and Zahodne et al’s study (Table 3). These may be due to: 16 of the 27 patients (59.3%) of the depressed PD patients were on antidepressant in Zahodne et al’s study while in the present study only 1/58(1.7%) depressed PD patient was on antidepressant [30].

**Table 3:** **Frequencies of common depressive symptoms among depressed PD patients; study subjects (58) Vs Zahodne et al. study (27), Black Lion Hospital and Zewuditu Memorial Hospital**

Common depressive symptoms	Our study		Zahodne et al. study		P value for difference
	Frequency	Proportion	Frequency	Proportion	
Sleep change	58	100%	22	81%	0.003
Sad mood	47	81%	15	56%	0.019
Decreased social involvement	57	98%	25	923%	0.236
Weight/appetite changes	44	76%	16	59%	0.132
Psychomotor agitation/retardation	44	76%	20	74%	1.0
Fatigue	58	100%	25	93%	0.098
Self outlook	50	86%	15	56%	0.003
Concentration difficulties/indecisiveness	43	74%	23	85%	0.283
Suicidal ideation	24	41%	5	19%	0.05

Depression is under-recognized in Ethiopian patients with PD. The present study show only one (1.7%) patient out of the 58 depressed PD patients was on antidepressant. This was way lower than in Zahodne et al’s study, 16 of the 27 patients (59.3%) of the depressed PD patients were on antidepressant [30]. Even though, not to this extent, diagnosis of depression in PD patients could be missed. In a study of 101 PD patients done in Baltimore, Maryland, USA, the treating neurologists failed to recognize depression. The prevalence of this condition as identified by the treating neurologist was lower: 21% while standardized testing showed depression in 44% of patients [15]. There is also high patient flow in the hospitals since these hospitals are the only teaching and referral hospitals having a Neurology clinics for a country of 80 plus millions peoples [45]. These may reduce patient-doctor interaction time and physicians may not enquire for depressive symptoms. The other main reason may be most of the symptoms of depression are common manifestation of PD [40]. In general this degree of treatment gap shows low index of suspicion among treating physicians in the two hospitals. Diagnostic evaluations were limited, but treatment was available, although expensive. In spite of the limitations, patients with movement disorders require and seek care in Ethiopia in proportions comparable to developed nations [45].

In the present study, there were no demographic or medical factors except annual income in the PD population that increased the risk of depression in PD patient. This finding may have been due to low study power which increased the risk of a Type II error. Studies to evaluate a larger number of Ethiopian PD outpatients are warranted to identify further risk factors for depression in this patient group.

## Conclusion

As depression is one of the most disabling symptoms of PD, the value of regular depression screening and treatment has been repeatedly emphasized [46]. However, very little is known about factors that influence depression severity or the nature of specific depression symptoms in PD. Depression occurred in 58(57.42%) of our patients. Out of these patients only 1/58(1.7%) was on antidepressants. The low index of clinician suspicion and under treatment of depression in PD patients is startling. The QIDS-C16 takes approximately 5 to 7 minutes to complete. It can be used as a screening tool for even busy clinics. Further studies are needed to confirm these findings, and to further increase our understanding of specific signs and symptoms of depression in the context of PD. Routine use of screening tools and subsequent treatment of depression in PD patients are warranted.

### Limitations

Co-morbid psychiatric symptoms, particularly anxiety, were not evaluated and may also have been common in this population. Only 23(22.8%) patients had previous imaging. This may affect the definitive diagnosis of PD since having imaging may exclude other causes of Parkinsonism. Another limitation of this study is the absence of a depression control group without PD. Thus, we cannot determine the degree to which the pattern of depressive symptoms discerned in this population is related to co-morbid PD. Although one previous study did not find differences between depressed individuals with PD compared to those without PD, [47] other studies have found differences between depressed patients with PD and those without PD. Another potential limitation is small sample size, which did not provide a strong study power to tell the difference between groups.

## Authors’ information

**Dawit Kibru Worku**, MD is a senior medical specialist (Neurologist) currently works as a University lecturer and clinician, Addis Ababa, Ethiopia

Co-authors:

**Yared Mamushet Yifru**, MD, Msc is a consultant in Internal medicine and neurology, a subspecialist in Headache medicine at Department of Neurology, Addis Ababa University, Addis Ababa, Ethiopia.

**Douglas G. Postels**, MD is an Associate Professor of Pediatric Neurology, Michigan State University: 2010-present

Field Volunteer, Médecins Sans Frontières, Lubutu, Democratic Republic of Congo and Port au Prince, Haiti, 2009–2010

Department Head of Neurology, Presbyterian Hospital, Albuquerque, New Mexico, 2003–2009

Staff Pediatric Neurologist, Ochsner Clinic, New Orleans, Louisiana, 1994–2001

**Fikre Enquselassie Gashe**, PhD is a renowned epidemiologist published more than fifty papers and received many awards for his work. Currently works at Addis Ababa University, Department of Community Health.

## Authors’ original submitted files for images

Below are the links to the authors’ original submitted files for images. Authors’ original file for figure 1 Authors’ original file for figure 2 Authors’ original file for figure 3 Authors’ original file for figure 4 Authors’ original file for figure 5 Authors’ original file for figure 6

## Abbreviations

BDI: Beck depression inventory
CT: Computerized tomography
DBS: Deep brain stimulation
DSM-IV: Diagnostic and Statistical Manual of Mental Disorders IV
GDS-15: Geriatric Depression Scale (15)
HRSD17: Hamilton Rating Scale for Depression 17 item version
IRB: Institutional Review Board
IDSC_30_: Inventory of Depressive Symptomatology Clinician-Rated(30)
MDD: Major Depressive Disorder
MRI: Magnetic Resonant Imaging
PD: Parkinson’s Disease
OR: Odds ratio
QIDS-C16: Quick Inventory of Depressive Symptomatology Clinician-Rated (16)
REM-Sleep: Rapid Eye movement sleep
UK: United Kingdom
