# Supplementary figures and images for: Prevalence of depression in Parkinson’s disease patients in Ethiopia
Source: J Clin Mov Disord. 2014 Dec 12;1:10. doi: 10.1186/s40734-014-0010-3 (PMC4711030; doi:10.1186/s40734-014-0010-3)

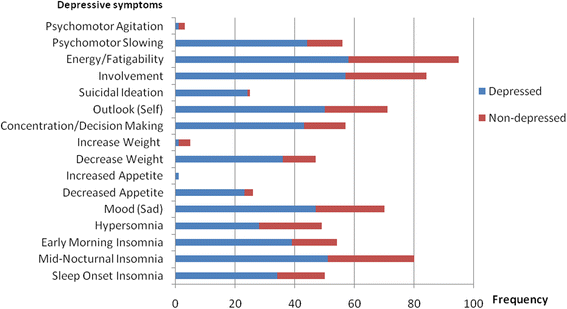

Supplement: Supplementary file 1 — Authors’ original file for figure 1 [file 40734_2014_10_MOESM1_ESM.gif]

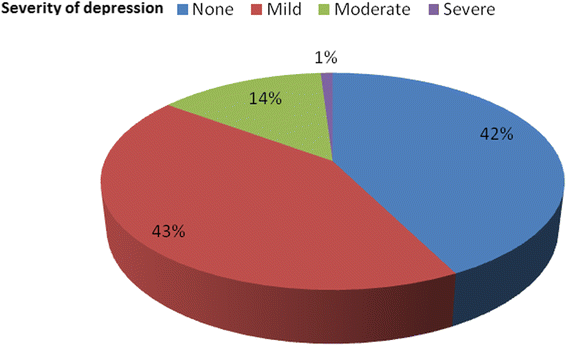

Supplement: Supplementary file 2 — Authors’ original file for figure 2 [file 40734_2014_10_MOESM2_ESM.gif]

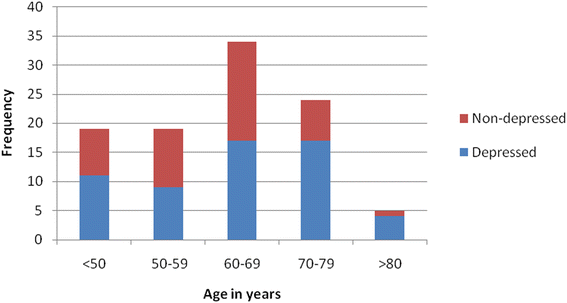

Supplement: Supplementary file 3 — Authors’ original file for figure 3 [file 40734_2014_10_MOESM3_ESM.gif]
