# Supplementary material for: Prevalence of depression in Parkinson’s disease patients in Ethiopia
Source: J Clin Mov Disord. 2014 Dec 12;1:10. doi: 10.1186/s40734-014-0010-3 (PMC4711030; doi:10.1186/s40734-014-0010-3)
Supplement: Supplementary file 4 — Authors’ original file for figure 4 [file 40734_2014_10_MOESM4_ESM.doc]

| Variables | Depressed | | Not Depressed | |  |
| --- | --- | --- | --- | --- | --- |
|  | Frequency | Proportion | Frequency | Proportion | P-Value |
| Handedness  Right  Left  Total | 56  2  58 | 96.6%  3.4% | 43  -  43 | 100  - | 0.5 |
| Age  <50  50-59  60-69  70-79  80 and above  Total  Mean+SD | 11  9  17  17  4  58  63.94+12.06 | 19.0%  15.5%  29.3%  29.3%  6.9% | 8  10  17  7  1  43  60.13+9.20 | 18.6  23.3  39.5  16.3  2.3 | 0.087 |
| Gender  Female  Male  Total | 18  40  58 | 31.0  69.0 | 13  30  43 | 30.2  69.8 | 1.0 |
| Marital Status  Married  Separated/divorced  Widowed  Total | 39  6  13  58 | 67.2  10.3  22.4 | 36  -  7  43 | 83.7  -  16.3 | 0.03  0.33 |
| Employment  Employed  Unemployed  Housewife  Retired  Out of job  Total | 12  46  10  20  16  58 | 20.7  79.3  21.7  43.5  34.8 | 14  29  7  14  8  43 | 32.6  67.4  24.1  48.3  27.6 | 0.25  1.0  0.84  0.81 |
| Education  No formal education  Primary  Secondary  More than secondary  Total | 29  18  6  5  58 | 50.0  31.0  10.3  8.6 | 13  10  12  8  43 | 30.2  23.3  27.9  18.6 | 0.80  0.02  0.06 |
| Monthly income (USD)  <300  300-600  600-900  >900  Total | 33  14  5  6  58 | 56.9  24.1  8.6  10.4 | 12  13  7  11  43 | 27.9  30.2  16.3  25.6 | 0.08  0.08  0.01 |
| Recent major life events  Yes  No  Total | 4  54  58 | 6.9  93.1 | 1  42  43 | 2.3  97.7 | 0.40 |
| Previous history of depression  Yes  No  Total | 2  56  58 | 3.4  96.6 | -  43  43 | -  100 | 1.0 |
| First degree family history of depression  Yes  No  Total | 3  55  58 | 5.2  94.8 | 2  41  43 | 5.2  94.8 | 1.0 |
| Age at onset  <50  50-59  60-69  >70  Total | 15  20  12  11  58 | 25.9  34.5  20.7  19.0  100 | 15  12  13  3  43 | 34.9  27.9  30.2  7.0  100 | 0.44  0.30  0.33 |
| Medication(s) taken/taking  Carbidopa-Levodopa  Trihexyphenidyl  Antidepressant | 54  29  1 | 40.3  21.6  1.7 | 35  19  2 | 49.3  26.8  4.7 | 0.12  0.69  0.57 |
