# Supplementary material for: Prevalence of depression in Parkinson’s disease patients in Ethiopia
Source: J Clin Mov Disord. 2014 Dec 12;1:10. doi: 10.1186/s40734-014-0010-3 (PMC4711030; doi:10.1186/s40734-014-0010-3)
Supplement: Supplementary file 5 — Authors’ original file for figure 5 [file 40734_2014_10_MOESM5_ESM.doc]

| Variables | Depressed | Non-depressed | Crude OR | P-Value |
| --- | --- | --- | --- | --- |
| Handedness  Right  Left  Total | 56  2  58 | 43  -  43 | ref  undetermined | 0.51 |
| Age  <50  50-59  60-69  70-79  80 and above  Total | 11  9  17  17  4  58 | 8  10  17  7  1  43 | 1.38(0.44-4.27)  0.9(0.30-2.77)  ref  2.43(0.80-7.35)  4.0(0.40-39.58) | 0.78  1.0  0.18  0.35 |
| Gender  Female  Male  Total | 18  40  58 | 13  30  43 | 1.04(0.44-2.45)  ref | 1.0 |
| Marital Status  Married  Separated/divorced  Widowed  Total | 39  6  13  58 | 36  -  7  43 | ref  undefined  1.71(0.62-4.78) | 0.03  0.33 |
| Employment  Employed  Unemployed  Total | 12  46  58 | 14  29  43 | 0.54 (0.22-1.33)  ref | 0.25 |
| Education  No formal education  Primary  Secondary  More than secondary  Total | 29  18  6  5  58 | 13  10  12  8  43 | ref  0.80(0.29-2.22)  0.22(0.07-0.73)  0.28(0.08-1.02) | 0.80  0.02  0.06 |
| Annual income(USD)  <300  300-600  600-900  >900  Total | 33  14  5  6  58 | 12  13  7  11  43 | ref  0.4(0.14-1.07)  0.26(0.07-0.98)  0.2(0.06-0.66) | 0.08  0.08  0.01 |
| Recent major life events  Yes  No  Total | 4  54  58 | 1  42  43 | 3.11(0.34-28.88)  ref | 0.39 |
| Previous history of depression  Yes  No  Total | 2  56  58 | -  43  43 | undefined  ref | 0.5 |
| First degree family history of depression  Yes  No  Total | 3  55  58 | 2  41  43 | 1.12(0.18-7.00)  ref | 1.0 |
| Age at onset  <50  50-59  60-69  >70  Total | 15  20  12  11  58 | 15  12  13  3  43 | 0.6(0.22-1.65)  ref  0.55(0.19-1.60)  2.2(0.51-9.51) | 0.44  0.30  0.33 |
| Medication(s) taken/taking  Carbidopa-Levodopa  Yes  No  Trihexyphenidyl  Yes  No  Antidepressant  Yes  No | 54  4 | 35  8 | ref  0.32(0.09-1.16) | 0.12 |
|  | 29  29 | 19  24 | 1.26(0.57-2.79)  ref | 0.69 |
|  | 1  57 | 2  41 | 0.36(0.03-4.10)  ref | 0.57 |
| Co-morbid medical condition(s)  Yes  No  Total | 21  37  58 | 9  34  43 | 0.19-1.16  ref | 0.12 |
