# Supplementary material for: Prevalence of depression in Parkinson’s disease patients in Ethiopia
Source: J Clin Mov Disord. 2014 Dec 12;1:10. doi: 10.1186/s40734-014-0010-3 (PMC4711030; doi:10.1186/s40734-014-0010-3)
Supplement: Supplementary file 6 — Authors’ original file for figure 6 [file 40734_2014_10_MOESM6_ESM.doc]

| Common depressive symptoms | Our study | | Zahodne et al study | | P value for difference |
| --- | --- | --- | --- | --- | --- |
|  | Frequency | Proportion | Frequency | Proportion |  |
| Sleep change | 58 | 100% | 22 | 81% | 0.003 |
| Sad mood | 47 | 81% | 15 | 56% | 0.019 |
| Decreased social involvement | 57 | 98% | 25 | 923% | 0.236 |
| Weight/appetite changes | 44 | 76% | 16 | 59% | 0.132 |
| Psychomotor agitation/retardation | 44 | 76% | 20 | 74% | 1.0 |
| Fatigue | 58 | 100% | 25 | 93% | 0.098 |
| Self outlook | 50 | 86% | 15 | 56% | 0.003 |
| Concentration difficulties/indecisiveness | 43 | 74% | 23 | 85% | 0.283 |
| Suicidal ideation | 24 | 41% | 5 | 19% | 0.05 |
